# Supplementary material for: MetaRibo-Seq measures translation in microbiomes
Source: Nat Commun. 2020 Jun 29;11:3268. doi: 10.1038/s41467-020-17081-z (PMC7324362; doi:10.1038/s41467-020-17081-z)
Supplement: Supplementary file 10 — Supplementary Data 7 [file 41467_2020_17081_MOESM10_ESM.zip › File2/Confidence_VeryHigh_Taxonomy/152387_out.krona.html]

Javascript must be enabled to view this page.

members
magnitude
magnitudeUnassigned
count
unassigned
taxon
rank

152387\_out

4

superkingdom
2
4

4
1239
phylum

186801
4
class

order
186802
4

family
4
31979

1485
4
genus

species
59620
4

SRS148721\_contig\_number\_16108SRS148784\_contig\_number\_29536SRS148874\_contig\_number\_12794SRS893230\_contig\_number\_contig-100\_3330.3331
